# Literature Review of Various Treatments in CNPAS and a Proposed Novel Clinical Treatment Algorithm

**DOI:** 10.3390/children12020250

**Published:** 2025-02-19

**Authors:** Omri Emodi, Nidal Zeineh, Ahmad Hija, Amir Bilder, Chaim Ohayon, Dekel Shilo, Miri Tzemach, Talia Gazit-Rappaport, Arie Gordin, Adi Rachmiel, Tal Capucha

**Affiliations:** 1 Department of Oral and Maxillofacial Surgery, Rambam Health Care Campus, Haifa 3109601, Israel; 2 Bruce Rappaport Faculty of Medicine, Technion—Israel Institute of Technology, Haifa 3200003, Israel; 3 Department of Orthodontics and Craniofacial Anomalies, Rambam Health Care Campus, Haifa 3109601, Israel; 4 Department of Otolaryngology-Head and Neck Surgery, Rambam Health Care Campus, Haifa 3109601, Israel

**Keywords:** congenital nasal pyriform aperture stenosis, maxillofacial deformity, treatment regimen, airway assessment, palatal expansion, orthodontic treatment

## Abstract

**Background:** Congenital nasal pyriform aperture stenosis (CNPAS) is a rare disorder characterized by a narrowed pyriform aperture and nasal obstruction. Children with CNPAS often exhibit a bony ridge on the underside of the secondary palate, a solitary central incisor, and a triangular-shaped palate. Due to its rarity, limited research exists, and there is no established treatment algorithm, which complicates management, particularly in craniofacial treatments and long-term follow-up. **Aims:** This study aimed to develop a comprehensive treatment algorithm based on long-term follow-up, focusing on maxillofacial deformities, dental considerations, and upper airway assessment. **Methods:** We conducted a retrospective study of four CNPAS patients treated at our institute. We performed a literature review, and a comparison was executed with our data in order to introduce our novel, age-specific clinical treatment framework. **Results:** A literature review was conducted, and different clinical parameters were examined. Previously published data were compared to our patients-related findings to develop our clinical treatment algorithm based on patients’ age. Patients were monitored for respiratory distress during the first two years of life. Children with cyclic cyanosis underwent surgical widening of the pyriform aperture through bony excess removal and nasal tubing to ensure airway patency. For those with ongoing desaturation events, neonatal palatal expansion was performed. At ages 10–12, additional evaluations using polysomnography and orthodontic assessments were conducted. Based on these findings, patients received surgically assisted rapid palatal expansion (SARPE) and further surgical widening of the pyriform aperture if obstructive sleep apnea (OSA) was present. Subsequent treatments included orthodontic care and restoration of the missing central incisor. **Conclusions:** We propose an age-based clinical treatment algorithm for CNPAS tailored to address individual patient needs throughout their growth.

## 1. Introduction

Congenital Nasal Pyriform Aperture Stenosis (CNPAS) is a rare craniofacial anomaly characterized by narrowing of the pyriform aperture and associated nasal obstruction. First identified in 1952, it was radiologically described in 1988, with the first clinical case published a year later [1,2,3]. This condition is diagnosed based on imaging criteria, with a pyriform aperture width of ≤11 mm in neonates considered the primary diagnostic threshold [4]. As the most anterior bony opening of the nasal airway, a narrowed pyriform aperture compromises the nasal breathing essential for neonatal survival, often presenting as respiratory distress.

CNPAS can present as an isolated condition or in association with other anomalies, including Holoprosencephaly (HPE), CHARGE syndrome, Velocardiofacial syndrome, or Solitary Median Maxillary Central Incisor (SMMCI) syndrome [5,6,7,8,9]. The defining anatomical features, including abnormal fusion of the lateral palatal shelves with the primary palate, result in a narrowed anterior nasal cavity [10,11]. Radiographically, a triangular palate, a bony ridge on the undersurface of the secondary palate, and a solitary central incisor are commonly observed [10]. Clinically, symptoms range from mild nasal obstruction to severe respiratory distress, tachypnea, difficulty feeding, and failure to thrive in neonates [12]. In severe cases, cyclic cyanosis and persistent oxygen desaturation may occur, necessitating urgent medical or surgical intervention [5,12]. The severity of symptoms and the condition’s rarity underscores the importance of timely diagnosis and individualized management strategies.

The diagnosis of CNPAS relies heavily on imaging modalities, with computed tomography (CT) scans serving as the gold standard for measuring pyriform aperture width and identifying associated craniofacial anomalies [4,10,12]. Magnetic resonance imaging (MRI) may supplement CT scans in complex syndromic cases [10]. Although advances in surgical techniques and postoperative care have improved outcomes, a notable gap remains in integrating patient age and clinical severity into treatment protocols. Despite extensive research, no study has provided a comprehensive algorithm incorporating age-specific management strategies for CNPAS.

In response to this gap, we propose a novel treatment algorithm designed to guide clinicians in managing CNPAS more effectively across different age groups and clinical severities. This algorithm aims to optimize treatment decisions and improve long-term outcomes by integrating both clinical and radiological findings.

This review synthesizes findings from the literature while introducing this novel, age-specific treatment framework. By addressing limitations in current protocols and providing a structured approach, this work aims to enhance the quality of care and improve the lives of children with CNPAS.

## 2. Materials and Methods

### 2.1. Literature Review

To explore the breadth of existing knowledge on Congenital Nasal Pyriform Aperture Stenosis (CNPAS), a systematic review of 25 peer-reviewed articles published between 2010 and 2024 was conducted. The search, performed exclusively on PubMed, focused on studies examining both conservative and surgical treatment modalities. Particular attention was given to identifying advancements, diagnostic criteria, and clinical outcomes relevant to the management of CNPAS.

The methods described in each article were analyzed and compared based on several key factors:

The number of patients treated using each technique.

The rationale for selecting specific treatments, including clinical severity or diagnostic thresholds.

The average pyriform aperture width was reported at the time of intervention.

Whether patient age was explicitly mentioned as a factor in treatment decisions.

The inclusion of follow-up data, particularly addressing the need for additional interventions or long-term outcomes.

The inclusion criteria encompassed:

Studies evaluating conservative and/or surgical management of CNPAS.

Articles reporting diagnostic thresholds, including pyriform aperture width ≤ 11 mm in neonates, consistent with accepted clinical standards [4].

Studies detailing treatment indications, procedural approaches, and postoperative outcomes.

Publications in English with clearly defined methodologies and results.

Exclusion criteria included:

Case reports or studies with insufficient methodological details.

**Figure 1:**
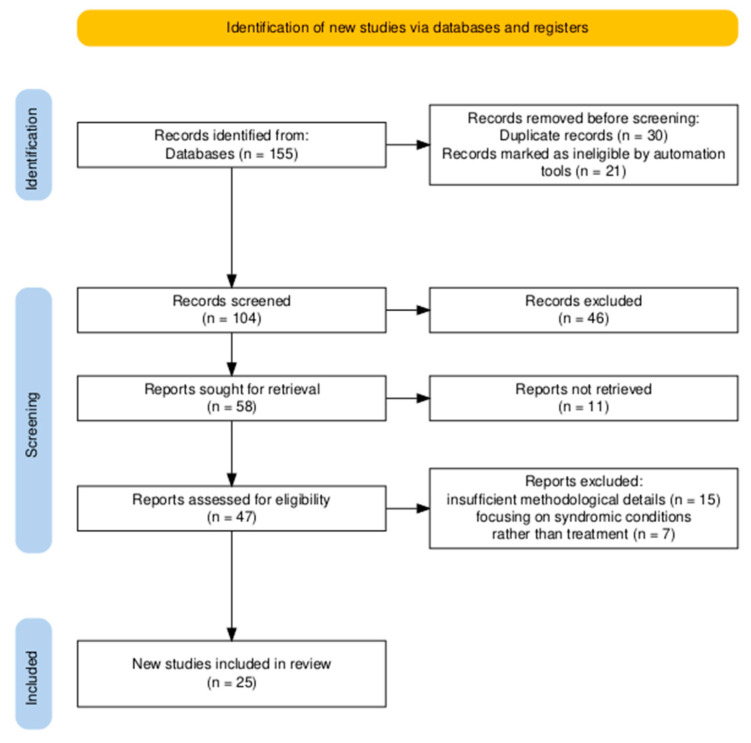
Flow Diagram for the systematic review.

Articles unrelated to CNPAS or focusing on syndromic conditions without specific reference to pyriform aperture management (Figure 1).

Two authors reviewed Each study independently to ensure consistency and reliability in data extraction. Discrepancies were resolved through mutual agreement. Key findings were synthesized to highlight trends in treatment, gaps in the literature, and areas for future research, particularly the integration of patient age into decision-making frameworks.

### 2.2. Retrospective Patient Data

In addition to the literature review, a retrospective analysis was conducted on four patients diagnosed with CNPAS and treated at our institute. Clinical data, including presenting symptoms, radiographic findings, treatment protocols, and outcomes, were reviewed to complement insights from the literature; Table 1 demonstrates a more detailed profile for each patient.

Cone beam computed tomography (CBCT) imaging was used preoperatively (T1) and six months post-treatment (T2) to assess structural changes. The imaging protocol ensured accuracy by:

Positioning patients in their natural head posture.

Minimizing motion artifacts by instructing patients to refrain from swallowing or breathing during scans.

Capturing a field of view extending from the orbit to the third cervical vertebra.

Three-dimensional reconstructions were generated using Dolphin Imaging 3D software (Dolphin Imaging Version 11.95 (SP3) Premium), and volumetric analyses were performed to measure the pyriform aperture and associated airway regions. These data provided a quantitative basis for evaluating treatment efficacy.

### 2.3. Algorithm Development

Insights from both the systematic review and retrospective patient data informed the development of a novel treatment algorithm. This algorithm addresses identified gaps in the literature, particularly integrating patient age and clinical severity into decision-making. The proposed framework outlines:

Conservative management for patients with mild symptoms.

Early surgical intervention for neonates exhibiting cyclic cyanosis or persistent respiratory distress.

Follow-up surgical and orthodontic interventions for patients aged 10–12 years, incorporating techniques such as surgically assisted rapid palatal expansion (SARPE) and pyriform aperture widening.

This structured approach is designed to optimize outcomes by tailoring interventions to the specific needs of patients at different stages of development.

### 2.4. Statistical Analysis

Quantitative data were analyzed using IBM SPSS Statistics for Windows, Version 25.0 (IBM Corp., Armonk, NY, USA). Numerical variables were compared using independent samples *t*-tests, with statistical significance defined as *p* < 0.05.

## 3. Results

### 3.1. Literature Review Findings

The systematic review analyzed 25 studies, comprehensively evaluating treatment modalities for Congenital Nasal Pyriform Aperture Stenosis (CNPAS). Treatment approaches varied widely, with decisions guided by clinical severity, pyriform aperture width, and postoperative considerations. Key findings are summarized in Table 2, outlining each method’s effectiveness and suitability for specific patient populations.

**Figure 2:**
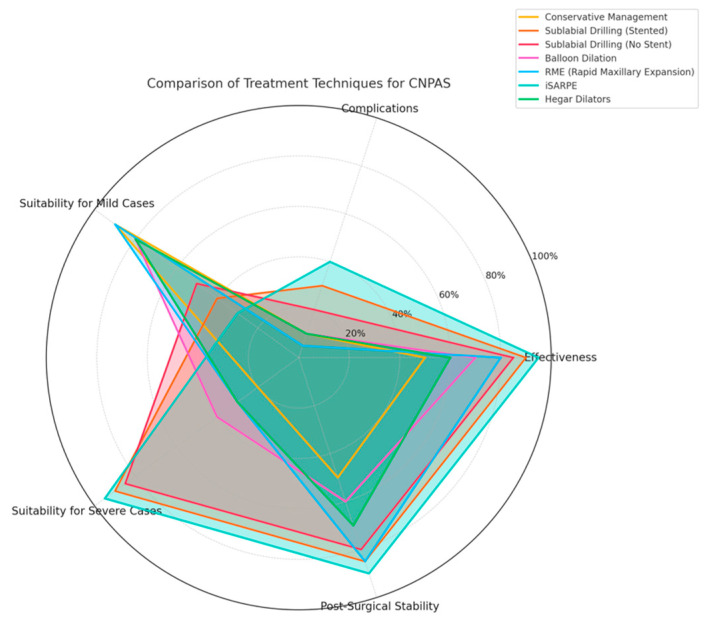
A Radar chart comparing treatment modalities for CNPAS, including conservative management, sublabial drilling, balloon dilation, RME, iSARPE, and Hegar dilators. Methods are evaluated across five parameters: effectiveness, suitability for mild and severe cases, post-surgical stability, and complications, highlighting their relative strengths and limitations.

Additionally, Figure 2 visualizes each method’s comparative indications, suitability, and outcomes, offering a multidimensional perspective on clinical applicability based on parameters such as effectiveness, post-surgical stability, and complications.

Conservative Management: Three studies highlighted conservative approaches as an initial strategy for mild cases with pyriform aperture widths ≥ 6 mm. Interventions such as nasal irrigation, decongestants, and anti-reflux therapy demonstrated high efficacy, with symptom resolution rates of 80–90% [13,14,15]. These measures delayed or avoided the need for surgical intervention in patients without significant respiratory distress. Figure 2 emphasizes the suitability of this approach for mild cases due to its high effectiveness and low complication rates.

Sublabial Drilling/Surgical: nine studies identified sublabial drilling as the gold standard for severe cases where pyriform aperture widths were <6 mm. This method achieved success rates of 85–100%, with postoperative stenting frequently employed to ensure airway patency and minimize restenosis risk [5,13,14,15,16,17,18,19,20] Sublabial drilling provided superior outcomes for neonates presenting with cyclic cyanosis or persistent desaturation compared to other methods. Figure 2 highlights sublabial drilling’s high effectiveness for severe cases, though its moderate risk of complications is noted.

Balloon-Assisted Dilation: Three studies described balloon-assisted dilation as a minimally invasive option for selected cases. Although its success rate (75%) was lower than that of sublabial drilling, balloon dilation was favored for its reduced recovery time and fewer postoperative complications. [12,21]. Figure 2 shows that balloon dilation is most suitable for borderline cases, balancing moderate effectiveness with minimal invasiveness.

Hegar or Gradual Dilation: Three studies reported on Hegar dilation, which provided symptom relief for patients with aperture widths of 5–7 mm. When combined with conservative management, this method delayed surgical intervention and offered a safe, effective alternative for selected cases [22,23]. Figure 2 positions Hegar dilation as an adjunctive method effective for borderline cases with moderate success rates and minimal complications.

Rapid Maxillary Expansion (RME): Three studies examined RME’s effectiveness for moderate cases, particularly those with aperture widths of ~6.3 mm. Aperture widening was achieved over 15–20 days, avoiding invasive procedures and demonstrating significant improvement in symptoms [23,24]. Figure 2 highlights RME’s noninvasive approach and its suitability for moderate cases with strong post-surgical stability.

Palatal Expansion (iSARPE): Two studies highlighted infant surgically assisted rapid palatal expansion (iSARPE) as a solution for severe cases with multilevel obstructions. This method achieved success rates of 87% and provided a comprehensive approach to expanding both the upper jaw and pyriform aperture [21,25]. As reflected in Figure 2, iSARPE demonstrates excellent effectiveness for severe cases with relatively fewer complications.

Combined Approaches: Two studies emphasized tailored treatment strategies, integrating multiple modalities to address complex anatomical presentations. These approaches achieved 85–90% success rates, with lower complication rates than standalone methods [26,27]. Figure 2 illustrates the versatility of combined approaches, which balance high effectiveness with reduced risks in complex cases.

### 3.2. Radiographic and Clinical Analysis

**Figure 3:**
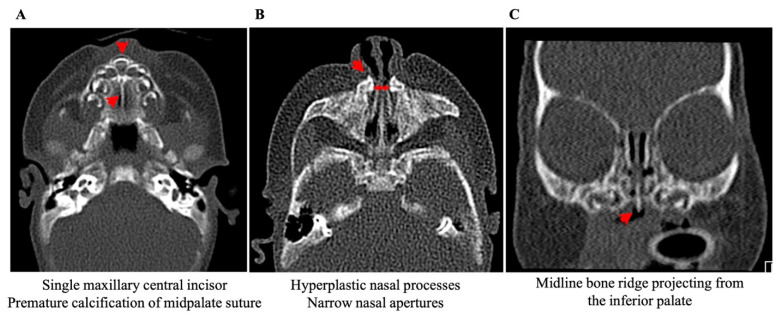
Demonstrates radiographic finding associated with CNAP, (**A**) SMCI and calcified midpalatal suture, (**B**) narrow nasal aperture and hyperplastic nasal process, and (**C**) midline inferior palatal ridge.

At our institute, four patients were diagnosed and treated for pyriform aperture stenosis. diagnosis was based on CT imaging findings and clinical symptoms, which included breathing difficulties, desaturation events, feeding issues, and resistance to nasogastric (NG) tube insertion. Radiographic imaging consistently revealed a single maxillary central incisor, Premature calcification of the mid-palatal suture, Hyperplastic nasal processes and narrow nasal apertures, and A midline bone ridge projecting from the inferior palate (Figure 3).

### 3.3. Treatment and Surgical Outcomes

**Figure 4:**
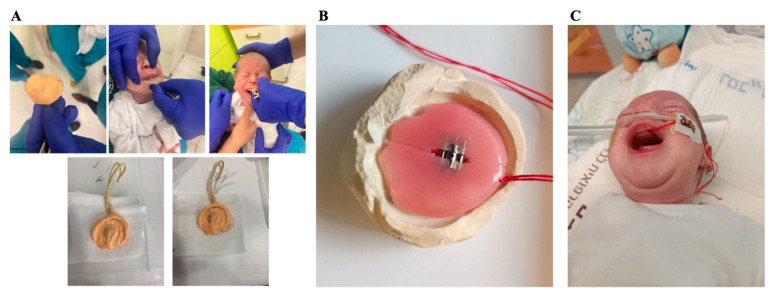
Demonstrates the process of Neonatal Palatal Expander Plate preparation, (**A**) impression taking, (**B**) the palatal expander, and (**C**) device application.

**Figure 5:**
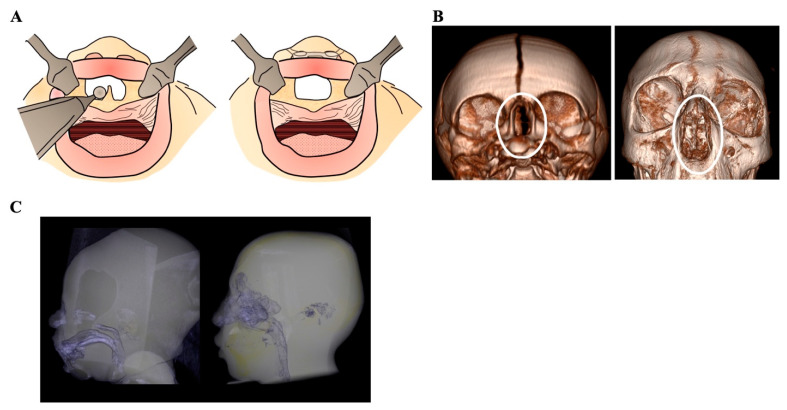
Illustrates (**A**) widening of the pyriform aperture by removal of the bony excess, (**B**) radiographic three-dimensional CT reconstruction demonstrating the increase in pyriform aperture (marked by white circle) following surgical widening and patient growth, and (**C**) demonstrates the airway enlargement with age.

Stage 1 Outcomes (0–24 months): Palatal expansion achieved a total widening of 12 mm over 32 days, with the screw rotated twice daily for 20 days, followed by one rotation per day for 12 days (Figure 4). Surgical widening of the pyriform aperture through a vestibular approach, combined with nasal tubing, resulted in substantial airway improvements. Follow-up CT scans demonstrated nasal floor expansion and narrowing elimination (Figure 5A–C).

**Figure 6:**
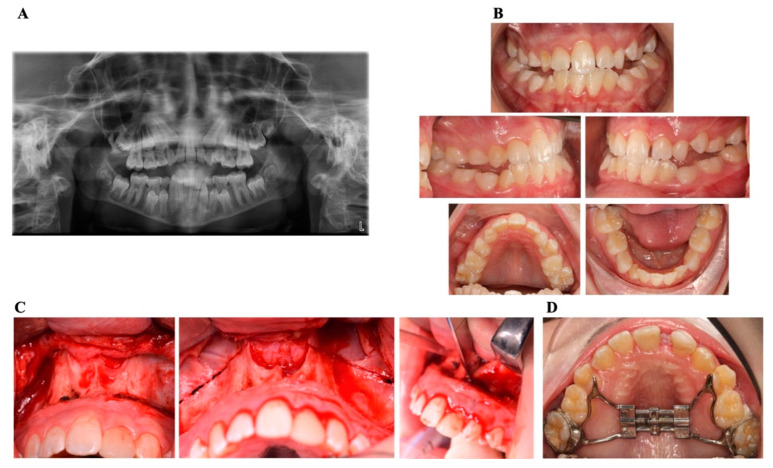
Demonstrates the (**A**) panoramic X-ray showing the single SMCI, (**B**) intra-oral images preoperatively, (**C**) intraoperative imaging of Le-Fort1 osteotomy for SARPE, and the (**D**) Hyrax device for palatal expansion.

**Figure 7:**
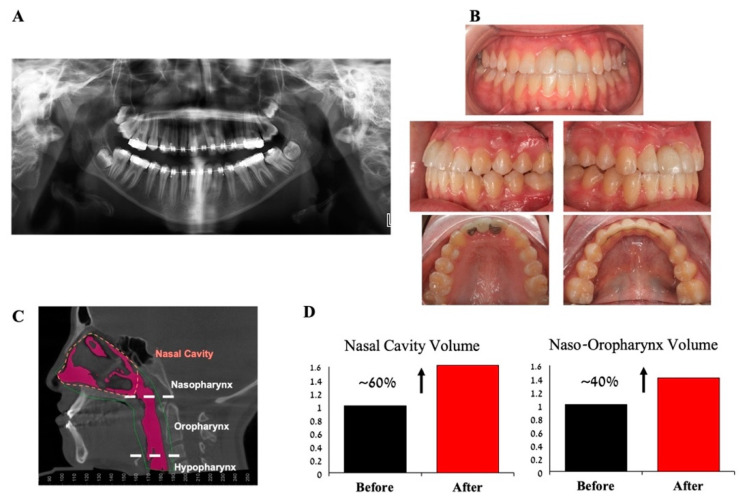
Demonstrates (**A**) panoramic X-ray of a patient showing a single central incisor, (**B**) clinical intraoral images with the use of Maryland bridge for the replacement of the missing incisor, (**C**) illustrates the airway volume of the patient, and (**D**) showing the change in the nasal cavity and nasopharynx volume change after surgery.

Stage 2 Outcomes (10–12 years): Mixed dentition patients demonstrating no improvement after Stage 1 underwent surgically assisted rapid palatal expansion (SARPE) to further widen the pyriform aperture. Orthodontic treatment aligned teeth and created space for missing central incisors. Final evaluations revealed restored occlusion, function, and aesthetics, ensuring a high quality of life (Figure 6). Airway segmentation analysis showed a 60% increase in nasal cavity volume and a 40% increase in naso-oropharynx volume compared to preoperative states (Figure 7).

### 3.4. Statistical Analysis

Table 3 presents the volumetric and structural changes in the oro-pharynx and nasal airway over time. The data include measurements at two-time points (T1 and T2) for both regions and aperture width changes. Statistical analysis confirms significant differences, emphasizing the importance of these changes in airway assessment.

## 4. Discussion

The rarity of Congenital Nasal Pyriform Aperture Stenosis (CNPAS) presents significant challenges for clinicians in terms of early diagnosis and establishing optimal treatment protocols. While upper airway obstruction in neonates affects approximately 1 in 5000 live births [4,28], and choanal atresia occurs in 1 in 8000 live births [4], the exact prevalence of CNPAS remains poorly defined [29]. In our retrospective series of four patients, we encountered varying clinical severities and ages at presentation—from neonates exhibiting cyclic cyanosis to older children with persistent dental and airway anomalies. This heterogeneity underscores the importance of individualized treatment strategies for CNPAS.

Thin-section computed tomography (CT) plays a pivotal role in diagnosis by revealing critical details such as overgrowth and medial displacement of the maxillary nasal processes. The literature broadly accepts a pyriform aperture width of less than 11 mm as a diagnostic threshold [30], but this parameter alone does not capture the full clinical variability of the condition. In our cohort, each patient underwent CT imaging to confirm the diagnosis and evaluate both the nasal aperture and associated findings (e.g., solitary median maxillary central incisor, midline inferior palatal ridge). Patient 1, for instance, presented with a narrow aperture measuring 5.8 mm, along with a noticeable midline bony ridge that contributed to respiratory compromise.

Clinical presentations of CNPAS typically reflect the degree of airway compromise and may include respiratory distress, feeding difficulties, and cyclic cyanosis. In the cohort analyzed here, the presence of solitary median maxillary central incisors underscores the known association with holoprosencephaly spectrum anomalies. Indeed, solitary incisors appear in 50–63% of CNPAS cases [31,32], reinforcing the importance of multidisciplinary evaluations aimed at detecting systemic associations that can influence both management and outcomes [33]. Two of our four patients (Patients 1 and 4) exhibited a single central incisor, necessitating close collaboration with pediatric dentistry and orthodontics. Both patients demonstrated characteristic nasal obstruction and had difficulties feeding, further underscoring the need for a swift, multidisciplinary approach to breathing, feeding, and orofacial development.

Early recognition is, therefore, crucial to mitigate not only immediate respiratory complications but also the broader consequences of this rare anomaly. For instance, Patient 1 was diagnosed in the neonatal period at 2 weeks old, which allowed for prompt surgical intervention. This early approach prevented complications such as prolonged hypoxia or failure to thrive, which can be especially damaging in neonates. In contrast, Patient 3, who presented at 10 years of age, experienced mild but chronic airway obstruction that was ultimately linked to suboptimal maxillofacial development.

Treatment strategies for CNPAS vary based on the severity of airway obstruction. Conservative approaches remain the first-line option for milder cases, with reported success rates of 80–90% through modalities such as nasal irrigation, decongestants, and anti-reflux therapy [13,14]. However, when conservative measures fail, or the obstruction is severe, surgical intervention becomes indispensable. In our series, Patient 2 initially attempted a conservative regimen with nasal decongestants and feeding modifications. However, persistent respiratory distress and recurrent desaturation events necessitated sublabial drilling and nasal stenting, which were pivotal in widening the nasal aperture and alleviating hypoxia.

Sublabial drilling, which is commonly referenced as the gold standard for pyriform aperture widths under 6 mm, achieves success rates of 85–100% [5,15], although postoperative stenting is often required to maintain adequate airway patency and reduce the likelihood of restenosis. Other methods, including balloon dilation, have shown moderate success in borderline cases [12,21], while adjunctive approaches such as Hegar dilation and rapid maxillary expansion can delay or lessen the need for more invasive procedures [22,23]. In Patients 1 and 2, sublabial drilling was performed to remove the excess bony ridge using a diamond bur, followed by careful placement of nasal stents to prevent restenosis. Patient 1 also underwent a neonatal palatal expansion, initiated within the first month of life. Over 32 days, the expansion protocol involved turning the screw twice daily for 20 days, then once daily for the remaining 12 days, resulting in an increase of the pyriform aperture from 5.8 mm preoperatively to 11.8 mm postoperatively. Oxygen saturation improved correspondingly, stabilizing at ~95% without supplemental oxygen.

**Scheme 1:**
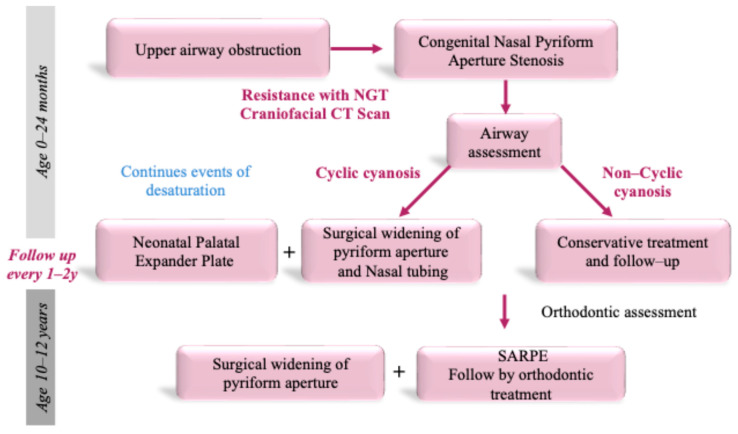
Illustrates the treatment sequence according to clinical and radiographic findings.

Notwithstanding these advances, there is a persistent need for systematic, age-specific treatment algorithms that address the varying demands of neonates, children, and adolescents. Few studies have proposed frameworks delineating the optimal timing and sequencing of interventions to achieve both immediate and long-term benefits, a gap that the proposed algorithm, outlined in Scheme 1, attempts to fill by emphasizing graded, developmentally tailored interventions. Our age-based protocol proved beneficial in older patients as well. Patients 3 and 4, both in mixed dentition (10 and 12 years old, respectively), underwent surgically assisted rapid palatal expansion (SARPE) after imaging confirmed persistent nasal obstruction and midface constriction. A Le Fort I–level osteotomy separated the palate, enabling controlled expansion with a Hyrax device. Patient 3 achieved a 60% increase in nasal cavity volume and a 40% increase in naso-oropharynx volume, as confirmed by postoperative cone beam computed tomography. Patient 4 had similar volumetric improvements and also benefited from orthodontic treatment to address dental malalignment and eventual restoration of the missing incisor.

Neonates presenting with non-cyclic cyanosis often benefit from conservative management, whereas those with cyclic cyanosis and continuous desaturation events may require prompt surgical widening of the pyriform aperture. Institutional data indicate that this early surgical approach can increase nasal cavity volume by 60% and naso-oropharynx volume by 40%, underscoring the clinical impact of timely intervention.

Our experience corroborates this, as Patient 1’s nasal cavity expansion followed a similar trajectory to the results observed in prior reports. By intervening early with sublabial drilling and palatal expansion, we significantly reduced respiratory distress and expedited a return to normal feeding and weight gain.

In mixed dentition, persistent functional and dental anomalies may necessitate surgical expansion of the palate in conjunction with further aperture widening and orthodontic adjustments, highlighting the importance of comprehensive, interdisciplinary care. Nevertheless, robust longitudinal studies evaluating these integrated strategies remain sparse, limiting our ability to formulate universally applicable guidelines.

Our integrated approach—combining maxillofacial, orthodontic, and otolaryngologic expertise—illustrates how a coordinated team can address both airway and craniofacial needs. However, we recognize the limited sample size of our case series and the importance of extended follow-up. Specifically, tracking patients beyond 24 months will be critical to confirm the durability of these airway improvements and determine if additional procedures or orthodontic refinements are necessary. Future investigations should also systematically measure parental and patient satisfaction to gauge quality-of-life outcomes, which remain understudied in this rare condition.

## 5. Conclusions

The proposed algorithm seeks to integrate current practices, incorporate emerging evidence, and guide future investigations that examine developmental and functional outcomes across the lifespan. Although further validation is necessary, situating immediate airway management within a broader therapeutic plan that addresses dentofacial development may offer meaningful improvements in short- and long-term prognoses for patients with CNPAS.

Future research should include larger, multicenter cohorts to validate our proposed treatment algorithm and explore variations in CNPAS presentation across diverse populations. Long-term, prospective studies will also be critical for assessing the stability of airway improvements, craniofacial outcomes, and quality of life. Further integration of patient- and parent-reported measures will help refine treatment protocols and optimize patient care.

## Figures and Tables

**Table 1:** Patients with CNPAS profile.

Patient	Age at Treatment	Diagnosis	Initial Symptoms	Previous Interventions	First Intervention	Second Intervention
*1*	11 years	Piriform aperture stenosis, nasal stenosis, single central incisor	Nasal obstruction, failed orthodontic expansion, skeletal discrepancy	Quad-Helix expansion (unsuccessful)	Planned SARPE with paramedian osteotomy	Follow-ups
*2*	Patient history spans from birth to 11 years old	Upper airway obstruction, adrenal insufficiency, progressive hearing loss	Cyanosis at birth, respiratory distress, sleep-related breathing difficulties	Neonatal surgery (incomplete correction), hearing aid placement	Conservative management, ENT and audiology follow-up; Late intervention: planned surgical airway expansion	Underwent SARPE at age 11; current follow-ups at age 14
*3*	3 years	Piriform aperture stenosis, single primary central incisor	Snoring, noisy breathing, recurrent respiratory infections	Nasal wall drilling for airway relief	Neonatal palatal expander planned	Possible future surgical correction if nasal obstruction worsens
*4*	20 days	Pyriform aperture stenosis, postoperative complications with nasal synechiae	Respiratory distress, feeding difficulty, desaturation episodes	Sub-labial PAS correction, nasal cannula placement	Scheduled for additional surgery to release nasal adhesions	Potential additional interventions depending on airway development

**Table 2:** Summary of reviewed treatment methods.

Method	Number of Articles	Average Aperture Width (mm)	Key Outcomes
Conservative Management	3	≥6 mm	Effective for mild cases; success rates of 80–90%.
Sublabial Drilling	9	<6 mm	Gold standard for severe cases; success rates of 85–100%.
Balloon-Assisted Dilation	3	Not specified	Promising minimally invasive alternative; moderate success rates (75%).
Hegar or Gradual Dilation	3	5–7 mm	Adjunctive method with significant symptom relief.
Rapid Maxillary Expansion	3	~6.3 mm	Effective for moderate cases; avoids surgery.
Palatal Expansion (iSARPE)	2	<6 mm	Addresses multilevel obstructions; high success rates (87%).
Combined Approaches	2	5.5–6 mm	Tailored to complex cases; success rates of 85–90%.

**Table 3:** Volume and aperture width changes in Oropharynx and Nasal airway.

Patient	Oro-Pharynx T1 (mm^3^)	Oro-Pharynx T2 (mm^3^)	ΔT (%)	Nasal Airway T1 (mm^3^)	Nasal Airway T2 (mm^3^)	ΔT (%)	Aperture Width (mm) T1	Aperture Width (mm) T2
1	6900	9598	39.1	5739	9125	59	8.3	13.9
2	9100	12,830	41	7451	11,996	61	6.6	13.7
3	5200	7238	39.2	4254	6636	56	3.6	9.1
4	4700	6627	41	3756	6159	64	4.9	9.8
Average	6475	9073.25	40.1	5300	8479	60	5.85	11.625
*p*-value			0.0083			0.0084		

## Data Availability

The data that support the findings of this study are available from the corresponding author upon reasonable request. The data are not publicly available due to privacy reasons.
